# Supplementary material for: Radiologist workforce challenges and the burden of image interpretation in Ghana: Perspectives of frontline doctors and implications for healthcare delivery
Source: PLOS Glob Public Health. 2026 Jun 3;6(6):e0006456. doi: 10.1371/journal.pgph.0006456 (PMC13232841; doi:10.1371/journal.pgph.0006456)
Supplement: Attachment — Submitted filename: Response to reviewers.docx [file pgph.0006456.s001.docx]

Thank you for insightful feedback and the opportunity to address your comments.

**Reviewers’ comments**

| **Comment** | **Response** |
| --- | --- |
| However, its impact is limited by a lack of empirical data to quantify the scale and severity of the problem it describes. The summary describes the findings but does not present the underlying data. A key weakness (e.g., actual average turnaround times, the frequency of report-less X-rays, rates of clinician-reported diagnostic uncertainty) to substantiate the powerful qualitative claims. All assertions about frequency and impact should be directly backed by the study's own data. Without knowing the population size at each of the participating hospitals, there is a risk that the findings, while likely representative, may be overgeneralized for the entire country or region. All assertions about frequency and impact should be directly backed by the study's own data. | We sincerely thank the reviewer for this thoughtful observation. We would like to respectfully clarify that this manuscript was designed as a qualitative descriptive study, employing semi-structured interviews and reflexive thematic analysis to explore clinicians' experiences and perceptions of radiologist shortages and their impact on image interpretation and patient care in Ghana.  Given the qualitative nature of the study, our aim was not to quantify the frequency or severity of the issue, but rather to capture in-depth, contextualised accounts from frontline medical doctors across multiple healthcare settings. Thematic analysis was used to derive meaning from participant narratives, not to generate statistical estimates or figures. Accordingly, the study does not present numerical data such as average turnaround times or frequencies of unreported X-rays, as this would fall outside the remit and methodological boundaries of qualitative research.  This study forms part of a larger four-phase, multi-sequential doctoral research study. Within this broader study, seven interrelated empirical sub-studies were conducted including this current manuscript. Quantitative findings including radiologist availability, average image interpretation turnaround times, annual X-ray examination volumes, and frequencies/volumes of unreported radiographs have been reported in separate peer-reviewed manuscript. To avoid redundancy and maintain focus on the qualitative strand, we have not duplicated those quantitative results here. However, in response to the reviewer’s concern, we have revised the introduction section to briefly incorporate relevant quantitative findings for context and to better situate the present study’s contribution. We have also further clarified this in the methods section. (see lines 76-88; lines 114-117). Thank you. |
| Justification for the sample size and the selection of participating hospitals | Thank you for the comment. This study employed a qualitative design grounded in exploratory inquiry, where the primary aim was to gain in-depth understanding of clinicians’ experiences and perceptions. In qualitative research, sample size is guided by the concept of information power (Malterud et al., 2016), with adequacy assessed by the richness of data in relation to the study’s aim, sample specificity, and quality of dialogue.  In this study, the final sample size was considered sufficient at the point of data saturation, where no new insights were emerging and the data had become repetitive across interviews (Saunders et al. 2017; Hennink & Kaiser 2022), indicating that saturation had been reached. This indicated that the data collected were both comprehensive and adequate to address the study objectives. We have revised the manuscript to include this.  Also, participants from health facilities were purposefully selected to reflect maximum variation in healthcare facility levels and geographic distribution across Ghana. This variation enabled a broader exploration of the issues across diverse resource settings, increasing the transferability of findings. Participants were selected based on their routine request and use of radiological examinations in patient management, aligning with the study’s focus on challenges associated with radiological interpretation.  The manuscript has been revised to include these (lines 129-134).  Thank you |
| The population sizes and clinical contexts of the participating hospitals should be provided to contextualize the findings and prevent overgeneralization for the entire country or region without sufficient evidence. | Thank you for the comment.  Ghana’s healthcare system is structured into four tiers. At the base is the primary healthcare level, comprising basic clinics and community-based services that serve as the first point of contact between patients and the formal health system. The second level (district hospitals) functions as the first referral facilities, while the regional level offers specialized medical services and supports the lower tiers. At the top is the tertiary level, which provides advanced, complex medical care and serves as the apex referral system within the country’s healthcare structure. The manuscript has been revised to include this for proper context and background. (see lines 121-128). Thank you |
| **Editorial comments** | |
| Please include more detail on how participants were selected/recruited in the methods section. | We have expanded the methodology section to clarify how participants were identified, recruited, and contacted in the study. Thank you |
| Please upload a copy of the interview guide & COREQ checklist as supplementary files. | Thank you, these have been added. |
| Please provide more detail on how the themes were developed. | Thank you, details have been provided in the revised manuscript. (see lines 161-171) |
| Include further discussion of the study limitations. | Thank you for the comment. The limitation section has been revised as suggested |
| If available, authors should provide the data requested by reviewer 1, or else at a minimum present more local/national/regional statistics in the Introduction/Discussion on the topics cited by the reviewer. | Thank you for the comment. This has been addressed in the revised manuscript. |
