# Supplementary material for: Radiologist workforce challenges and the burden of image interpretation in Ghana: Perspectives of frontline doctors and implications for healthcare delivery
Source: PLOS Glob Public Health. 2026 Jun 3;6(6):e0006456. doi: 10.1371/journal.pgph.0006456 (PMC13232841; doi:10.1371/journal.pgph.0006456)
Supplement: Attachment — Submitted filename: Review report.docx [file pgph.0006456.s002.docx]

**Peer review report**

**Manuscript #:** PGPH-D-25-02975R1

Thank you for the opportunity to review this interesting research. The manuscript has the potential to make a valuable and engaging contribution. However, several aspects of the work require further clarification and development before it is suitable for publication.

**Title:** The title effectively reflects the topic and its implications; however, it does not identify the study population. Clarify whether the perspectives or experiences are those of radiologists, radiographers, or clinicians, and incorporate this information into the title.

**Abstract**

- State whether participants are from the public, private, or both sectors, as this could influence outcomes.
- Please ensure consistency in terminology. In the aim, the term frontline clinicians (line 39) is used, whereas in the conclusion, front-line medical practitioners appear (Line 53). This inconsistency may confuse readers.
- Under the results line 224, some participants suggested the need to introduce reporting radiographers like other Western countries and in Africa, Uganda and Zimbabwe (Approved in November 2025). This is an important point which is missing in the abstract.
- Keywords should be arranged in alphabetical order (Line 57).

**Introduction**

- **Line 82:** The term “radiographers” is used; however, some are reporting radiographers trained in clinical reporting. Please be specific.
- The aim stated in the abstract does not align with the aim in the introduction. The abstract uses the verb “explore”*,* whereas the introduction uses “examine”*.* The research aim should be stated consistently throughout the paper. The research aim should be more explicitly stated at the end of the introduction.
- Please clarify whether image interpretation **by referring clinicians** in Ghana is limited to plain radiography (X-rays) or also includes advanced imaging modalities. This distinction should be clearly stated. This can be made in the introduction or discussion sections of the manuscript.
- Important contextual information appears to be missing regarding the education and training in radiology of referring clinicians. Including details on medical doctors’ training and experience in radiology and image interpretation, both theoretical and practical, would strengthen the argument. This may include time allocated to radiology training. Additionally, the shortage of radiologists may result in greater emphasis on clinical service provision (reporting) than on clinical teaching, which is relevant to the study context. This may affect future clinicians.

**Materials and methods**

- State whether participants are from the public, private, or both sectors, as this could influence outcomes.
- The description of the recruitment process lacks sufficient detail, particularly regarding sampling strategies. Please clarify how participants were selected.
- It is unclear on what basis the interview guide was developed.
- The claim of data saturation is not justified. Provide more details. E.g., contacting additional interviewees to avoid early termination of data collection.
- The explanation of data analysis is incomplete. Please briefly outline all five relevant steps of Braun and Clarke’s six-phase thematic analysis framework. Additionally, clarify whether transcription and coding were conducted manually or using electronic tools.
- Data collection and data analysis should be presented as distinct sections. Consider writing about each in separate paragraphs for clarity.
- Trustworthiness strategies are not sufficiently described beyond peer debriefing. Include additional methods used to enhance credibility, dependability, confirmability, and transferability. You may use a table.

**Results**

- Line 134 does not address demographics but rather emerging themes. This content should be presented in a separate paragraph for clarity and logical flow.
- **T**he themes are well presented; however, the supporting explanations are limited. Expanding the narrative would help readers better understand the findings and follow the progression of ideas.
- Consider presenting sub-theme names explicitly and consistently in the results section. For example: **Sub-theme 1**: Frequency of Imaging Requests. This approach would improve structure and readability.

**Discussion**

- Begin the discussion by restating the aim of the study to orient readers and frame the interpretation of the findings.
- **Lines 289 to 300-** This section focuses on the establishment of reporting radiographers in Ghana. Consider strengthening the discussion by providing examples from other settings where this practice has been implemented and outlining its benefits. For instance, in November 2025, the scope of practice for radiographers in Zimbabwe was expanded to include clinical reporting, making it the second country in Africa, after Uganda, to implement this model. Additionally, countries such as South Africa, Zambia and Nigeria are currently exploring the establishment of reporting radiographers. Including such comparisons would provide regional context and support the relevance of your findings.
- Education and training in radiology within medical schools require further discussion, as this appears to be a central issue. Consider comparing your findings with evidence from studies conducted outside Ghana to highlight similarities or differences in training experiences, competency development, and clinical preparedness.

**Conclusion**

- Among the strategies is the establishment of reporting radiographers. This is important to the development of quality imaging services.
