# Supplementary material for: Radiologist workforce challenges and the burden of image interpretation in Ghana: Perspectives of frontline doctors and implications for healthcare delivery
Source: PLOS Glob Public Health. 2026 Jun 3;6(6):e0006456. doi: 10.1371/journal.pgph.0006456 (PMC13232841; doi:10.1371/journal.pgph.0006456)
Supplement: Attachment — Submitted filename: Response to reviewers updated new.docx [file pgph.0006456.s003.docx]

Thank you for the feedback.

The comments were very helpful in strengthening the clarity, structure, and transparency of the paper.

|  | **Response** |
| --- | --- |
| **Reviewer 1** |  |
| Thank you for addressing all the comments and additional clarification. | Thank you |
| **Reviewer 2** |  |
| The title effectively reflects the topic and its implications; however, it does not identify the study population. Clarify whether the perspectives or experiences are those of radiologists, radiographers, or clinicians, and incorporate this information into the title. | The title has been revised to explicitly reflect the study population (frontline doctors). |
| Abstract  State whether participants are from the public, private, or both sectors, as this could influence outcomes. | This has been clarified in the abstract, thank you |
| Abstract  Please ensure consistency in terminology. In the aim, the term frontline clinicians (line 39) is used, whereas in the conclusion, front-line medical practitioners appear (Line 53). This inconsistency may confuse readers. | Thank you. Clinician in this context refers to medical doctors. This has been corrected |
| Under the results line 224, some participants suggested the need to introduce reporting radiographers like other Western countries and in Africa, Uganda and Zimbabwe (Approved in November 2025). This is an important point which is missing in the abstract. | Thank you for the suggestion. Radiographer reporting currently falls outside the scope of practice of radiographers in Ghana. The medical doctors rather suggested the need to involve trained radiographers in preliminary image evaluation (PIE). This has been captured in the revised abstract. |
| Keywords should be arranged in alphabetical order (Line 57). | This has been corrected |
| Introduction  Line 82: The term “radiographers” is used; however, some are reporting radiographers trained in clinical reporting. Please be specific. | We acknowledge that in some countries radiographers are trained and authorised to perform formal clinical reporting. However, in the Ghanaian context radiographer reporting is not currently within the professional scope of practice. The reference to radiographers in this section relates to informal assistance with image interpretation or participation in preliminary image evaluation (PIE), rather than formal diagnostic reporting. To avoid ambiguity, this clarification has now been added to the manuscript. |
| The aim stated in the abstract does not align with the aim in the introduction. The abstract uses the verb “explore”, whereas the introduction uses “examine”. The research aim should be stated consistently throughout the paper. The research aim should be more explicitly stated at the end of the introduction. | Thank you for this helpful observation. The manuscript has been revised to ensure consistency in the wording of the study aim across the abstract and introduction. The verb “explore” is now used consistently throughout. |
| Please clarify whether image interpretation by referring clinicians in Ghana is limited to plain radiography (X-rays) or also includes advanced imaging modalities. This distinction should be clearly stated. This can be made in the introduction or discussion sections of the manuscript. | In most healthcare facilities in Ghana, this clinician-led interpretation predominantly relates to plain radiography, as access to advanced imaging modalities such as CT or MRI is more limited and typically interpreted by radiologists. It is also important to note that the interpretation challenges described in this study largely relate to plain radiography, which constitutes the most commonly available imaging modality in many Ghanaian healthcare facilities. This has been clarified in the discussion section. |
| Important contextual information appears to be missing regarding the education and training in radiology of referring clinicians. Including details on medical doctors’ training and experience in radiology and image interpretation, both theoretical and practical, would strengthen the argument. This may include time allocated to radiology training. Additionally, the shortage of radiologists may result in greater emphasis on clinical service provision (reporting) than on clinical teaching, which is relevant to the study context. This may affect future clinicians. | Thank you for this valuable suggestion. A paragraph has been added to the introduction describing the typical structure and scope of radiology training in medical education, including the limited duration of radiology exposure and the potential impact of radiologist workforce shortages on clinical teaching and supervision. This addition strengthens the contextual background and clarifies why clinicians may experience challenges in image interpretation when radiologist support is limited. |
| Methods  State whether participants are from the public, private, or both sectors, as this could influence outcomes. | Thank you for this helpful suggestion. The Methods section has been revised to provide greater detail regarding the recruitment and sampling strategy. |
| It is unclear on what basis the interview guide was developed. | Thank you. The development of the interview guide was already described in the Methods section as being informed by the study objectives and relevant literature. To further improve clarity and transparency, we have revised the description to more explicitly state the basis for the development of the interview guide and the key topic areas covered. |
| The claim of data saturation is not justified. Provide more details. E.g., contacting additional interviewees to avoid early termination of data collection. | Thank you. The manuscript already described the use of information power and data saturation to guide sample adequacy. To further clarify this process, we have expanded the Methods section to explain that data collection and analysis occurred concurrently, allowing the research team to assess the emergence of new concepts during the interview process. Recruitment continued until no new themes or insights emerged from additional interviews, indicating that thematic saturation had been achieved. |
| The explanation of data analysis is incomplete. Please briefly outline all five relevant steps of Braun and Clarke’s six-phase thematic analysis framework. | Thank you for this helpful comment. However, we respectfully argue that when a paper cites Braun & Clarke properly, it is not deemed necessary to restate all six steps, because they are already well established in the literature. The manuscript already described the use of Braun and Clarke’s six-phase thematic analysis framework and indicated that NVivo 14 software was used to support data organisation and coding.  However, to further improve clarity, we have revised the description of the analytical process to more explicitly summarise the stages of the framework while maintaining conciseness. |
| Additionally, clarify whether transcription and coding were conducted manually or using electronic tools | This has been clarified, thank you |
| Data collection and data analysis should be presented as distinct sections. Consider writing about each in separate paragraphs for clarity. | Thank you for the feedback. The manuscript has been revised accordingly |
| Trustworthiness strategies are not sufficiently described beyond peer debriefing. Include additional methods used to enhance credibility, dependability, confirmability, and transferability. You may use a table. | Thank you for this helpful suggestion. However, several strategies to enhance the trustworthiness of the findings are already embedded across several sections of the Methods, including purposive sampling, prolonged engagement with the data, member checking, peer debriefing, and transparent documentation of the analytical process.  For instance:  Purposive sampling and maximum variation support transferability  Member checking and prolonged engagement support credibility  Peer debriefing and team discussions support dependability  Transparent analytic procedures and NVivo coding support confirmability  We hope that this addresses the concern raised.  Thank you |
| Results  Line 134 does not address demographics but rather emerging themes. This content should be presented in a separate paragraph for clarity and logical flow. | Thank you for the helpful observation. The Results section has been revised to improve clarity and logical flow by separating the description of participant characteristics from the presentation of the thematic findings. Participant demographics are now presented in a distinct paragraph before introducing the themes identified during analysis. |
| The themes are well presented; however, the supporting explanations are limited. Expanding the narrative would help readers better understand the findings and follow the progression of ideas. | Thank you. The section has been slightly revised to ensure it is not repetition of information provided in the discussions. |
| Consider presenting sub-theme names explicitly and consistently in the results section. For example: Sub-theme 1: Frequency of Imaging Requests. This approach would improve structure and readability | Thank you for this important feedback, this has been corrected |
| Discussion  Begin the discussion by restating the aim of the study to orient readers and frame the interpretation of the findings. | Thank you, the discussion section has been revised to begin with a brief restatement of the study aim and a summary of the key findings to better orient readers and frame the interpretation of the results. |
| **Lines 289 to 300-** This section focuses on the establishment of reporting radiographers in Ghana. Consider strengthening the discussion by providing examples from other settings where this practice has been implemented and outlining its benefits. For instance, in November 2025, the scope of practice for radiographers in Zimbabwe was expanded to include clinical reporting, making it the second country in Africa, after Uganda, to implement this model. Additionally, countries such as South Africa, Zambia and Nigeria are currently exploring the establishment of reporting radiographers. Including such comparisons would provide regional context and support the relevance of your findings. | Thank you for the suggestion. We agree that regional comparisons provide useful context.  In response, we have expanded the discussion to include regional developments in radiographer role extension. While there are emerging reports of clinical reporting initiatives in countries such as Uganda and Zimbabwe, as well as ongoing discussions in South Africa, Zambia, and Nigeria, the available literature remains limited and, in some cases, lacks detailed documentation of fully implemented clinical reporting systems. As such, we have taken a cautious approach in interpreting these developments.  We have however acknowledged these developments in the revised manuscript noting that, these regional trends highlight a broader recognition of the potential role of radiographers in supporting diagnostic pathways, particularly in contexts affected by radiologist workforce shortages. |
| Education and training in radiology within medical schools require further discussion, as this appears to be a central issue. Consider comparing your findings with evidence from studies conducted outside Ghana to highlight similarities or differences in training experiences, competency development, and clinical preparedness. | Thank you very much. We have revised the manuscript accordingly |
| Conclusion  Among the strategies is the establishment of reporting radiographers. This is important to the development of quality imaging services. | Thank you. We have revised the manuscript accordingly |
| **Reviewer 3** |  |
| A good point is made regarding the sufficiency of the sample size and this does not need to be changed for this article, however, consideration might be given in future to the sub divisions of the sample and considering the sufficiency of the sample within these. | While the current study was not designed to support subgroup-level analysis, we agree that this represents an important consideration for future research. This has been noted in the limitations section to guide subsequent studies. |
| Further detail on the researchers’ approach would be useful – how many authors generated initial codes? What was the procedure in cases of discrepancy? | Additional detail has been included in the Methods section to clarify the coding process, including the number of researchers involved in initial coding and the approach used to resolve discrepancies through discussion and consensus. Thank you |
| Was there an impartial researcher also who was not involved in the interviews? Were the interviews conducted with one researcher present? Were any field notes taken to support transcription? | Thank you  Interviews were conducted by a single member of the research team, which facilitated consistency in data collection and rapport with participants. The interviews were conducted via phone, and no additional researchers were present to minimise potential influence on participants’ responses.  Given the virtual format, formal field notes were not taken. However, brief reflective notes were documented following each interview to capture key reflections and nuances in participants’ responses. |
| It would be useful to have the script available as part of the paper also. | Thank you for this comment. We understand the importance of transparency in qualitative research. However, full verbatim interview transcripts cannot be made publicly available due to ethical considerations and participant confidentiality, as the data contain potentially identifiable information. |
| Were there differences found in the perspectives of the various levels of experience of the doctors? It would be very useful to have a table to indicate the code and the year of experience of the clinician, however as there is only one interviewee in one experience band, I understand that this might not be appropriate. | Thank you. We agree that examining differences across levels of clinical experience could provide valuable additional insights. However, given the relatively small sample size and the uneven distribution of participants across experience levels, subgroup analysis was not considered methodologically appropriate, as it may risk overinterpretation of the data. |
| Comments from the previous review have been addressed and there has been significant changes made to the original submission. | Thank you |
